# Increased Lamin B1 Levels Promote Cell Migration by Altering Perinuclear Actin Organization

**DOI:** 10.3390/cells9102161

**Published:** 2020-09-24

**Authors:** Andrea Fracchia, Tal Asraf, Mali Salmon-Divon, Gabi Gerlitz

**Affiliations:** 1 Department of Molecular Biology, Faculty of Life Sciences and Ariel Center for Applied Cancer Research, Ariel University, Ariel 40700, Israel; andrea.fracchia01@gmail.com (A.F.); talbh92@gmail.com (T.A.); malisa@ariel.ac.il (M.S.-D.); 2 Adelson School of Medicine, Ariel University, Ariel 40700, Israel

**Keywords:** cell migration, actin, lamin A, lamin B1, nuclear envelope, LINC complex

## Abstract

Cell migration requires reposition and reshaping of the cell nucleus. The nuclear lamina is highly important for migration of both primary and cancer cells. B-type lamins are important for proper migration of epicardial cells and neurons and increased lamin B to lamin A ratio accelerates cancer cell migration through confined spaces. Moreover, a positive association between lamin B1 levels and tumor formation and progression is found in various cancer types. Still, the molecular mechanism by which B-type lamins promote cell migration is not fully understood. To better understand this mechanism, we tested the effects of lamin B1 on perinuclear actin organization. Here we show that induction of melanoma cell migration leads to the formation of a cytosolic Linker of Nucleoskeleton and Cytoskeleton (LINC) complex-independent perinuclear actin rim, which has not been detected in migrating cells, yet. Significantly, increasing the levels of lamin B1 but not the levels of lamin A prevented perinuclear actin rim formation while accelerated the cellular migration rate. To interfere with the perinuclear actin rim, we generated a chimeric protein that is localized to the outer nuclear membrane and cleaves perinuclear actin filaments in a specific manner without disrupting other cytosolic actin filaments. Using this tool, we found that disruption of the perinuclear actin rim accelerated the cellular migration rate in a similar manner to lamin B1 over-expression. Taken together, our results suggest that increased lamin B1 levels can accelerate cell migration by inhibiting the association of the nuclear envelope with actin filaments that may reduce nuclear movement and deformability.

## 1. Introduction

The nuclear envelope separates the nucleus from the cytoplasm. The nuclear envelope is composed of two membranes: the outer nuclear membrane (ONM), which faces the cytoplasm, and the inner nuclear membrane (INM), which faces the inside of the nucleus [1,2]. The outer nuclear membrane can interact with various cytoplasmic cytoskeletal elements including actin filaments, microtubule motor proteins, and intermediate filaments-interacting proteins. One of the major components that facilitate this interaction is the Linker of Nucleoskeleton and Cytoskeleton (LINC) complex that is composed of the INM proteins Sun1 and 2 and the ONM proteins Nesprins 1–3 [3,4]. In addition to the membranes, the nuclear envelope contains nuclear pore complexes (NPCs) to facilitate exchange of molecules with the cytoplasm [5] and in metazoans the INM is connected to a filamentous network termed the nuclear lamina [6,7,8].

The nuclear lamina is composed mainly of type V intermediate filament proteins termed lamins, which are classified into A- and B-type. A-type lamins are mainly expressed in differentiated cells from the *LMNA* gene that undergoes alternative splicing to form lamin A and lamin C. B-type lamins are ubiquitously expressed from the genes *LMNB1* and *LMNB2* to form the two main isoforms lamin B1 and B2, respectively. Lamins are key structural components of the nucleus that determine its shape, size, and mechanostability [6,9]. In addition, lamins are also important for chromatin organization, transcriptional control, DNA damage repair, DNA replication, cell division, and cell signaling [7,10,11].

B-type lamins are known to support cell migration during development: *Lmnb1* or *Lmnb2* knock-out mice have brain development impairs due to poor migration of neurons to form the correct layers of the cortex [12,13], while loss of lamin B1 farnesylation leads to detachment of chromatin from the nuclear envelope during neuronal migration [14]. Lamin B1 is also important for proper migration of mouse cardioepithelial cells during heart development [15]. However, analyses of cancer cell migration identified a migration inhibitory role for lamin B1 in lung cancer cells [16], while a migration promoting activity was found for lamin B1 in pancreatic cancer cells [17]. Lamin A analyses found that increased levels of lamin A inhibit cancer cell migration [18,19,20,21,22], while reduced lamin A levels accelerate cell migration rate [23]. In addition, a reduced ratio of lamin A to lamin B was found to accelerate the migration rate of cells while increasing the frequency of nuclear envelope rupture [19,24]. The major mechanism by which lamins affect cell migration is thought to be by altering the stiffness of the nuclear envelope: A-type lamins are thought to generate a stiffer filamentous network than B-type lamins [19,24,25,26,27,28,29,30]. In addition, epigenetic effects of lamin B1 were suggested to cause its inhibitory effect on lung cancer cell migration [16].

To evaluate the effects of lamins on melanoma cell migration and to identify additional mechanisms by which lamins can affect this process, we tested the effects of lamin A and lamin B1 over-expression on melanoma cell migration and on the perinuclear actin organization. In recent years, several perinuclear actin structures have been identified in migrating cells: the actin cap is composed of dorsal longitudinal actin filaments anchored to focal adhesion points at both of their ends while their central part is attached to the dorsal side of the nucleus by the LINC complex [31,32]. Transmembrane Actin-associated Nuclear (TAN) lines are dorsal actin filaments that are perpendicular to the leading edge of the cell. They are pushed towards the trailing edge of the cell while interacting with the dorsal nuclear side through the LINC complex to pull the nucleus backward to establish cellular polarization [33,34]. Both actin structures are LINC complex-dependent and were found in primary and immortalized cells, but not in cancer cells. In addition, Arp2/3-dependent actin filaments were found to nucleate around the nucleus of primary dendritic cells while migrating in a confined environment to disrupt the nuclear lamina [35].

Here, we show that over-expression of lamin B1 but not of lamin A enhanced melanoma cell migration in parallel to disruption of a perinuclear actin rim. The perinuclear actin rim was found in melanoma cells that were induced to migrate in the wound healing assay or were constrained by a collagen matrix. This perinuclear actin rim was a long-lived actin structure in migrating cells. To detect the effect of this actin rim on cell migration rate, we generated a chimeric protein by fusing a modified fragment of Nesprin 2 (KASH2ext) to the catalytic domain of the actin severing protein Gelsolin (GSN). This chimeric protein disrupted the defined perinuclear actin rim, leading to enhanced cellular migration rate in both the wound healing assay and the Transwell assay. Whereas, interference with the LINC complex did not alter either the perinuclear actin rim or the cellular migration rate. Thus, an alternative mechanism to the LINC complex may link the nuclear lamina to perinuclear actin filaments, enabling lamin B1 to accelerate cell migration rate by disrupting the perinuclear actin rim.

## 2. Materials and Methods

### 2.1. Datasets

Normalized expression levels (RSEM counts) of *LMNB1* and *LMNA* genes in normal tissues from GTEx and tumor samples from the TCGA datasets, together with sample type classification (normal tissue, normal tissue adjacent to a tumor, primary tumor, metastasis), were downloaded from the Xena browser [36]. Boxplots were created using the R programming language.

### 2.2. Cell Culture

Mouse melanoma B16–F10 cell line purchased from ATCC (Manassas, VA, USA) was grown as described previously [37].

### 2.3. Plasmids

Plasmids expressing lamin A and lamin B1 fused to GFP were a kind gift from Tom Misteli [38] and pEGFP-KASH2 and pEGFP-KASH2ext were a kind gift from Didier Hodzic [39]. Gelsolin was cloned by RT-PCR: for reverse transcription, total RNA was isolated from B16–F10 by the NucleoZOL kit (740404.200, MACHEREY-NAGEL, Düren, Germany) according to the manufacturer’s instructions and reverse-transcribed using GoScript™ Reverse Transcription System (Promega) and the oligonucleotide 5′-GAGAACTGAAACCTGGGT-3′. The N-terminal part of Gelsolin (aa 1–370) was amplified by PCR using KOD Hot Start DNA Polymerase (71086, Merck, Kenilworth, NJ, USA) and the oligonucleotides 5′-cagctagccaccATGGTGGTGGAGCACCCC-3′ and 5′-gcaccggtaTGGGGTACTGCATCTTGGAGAT-3′. The PCR product was ligated into NheI-AgeI sites in pEGFP-KASH2ext upstream to GFP to generate pGSN-EGFP-KASH2ext. Transfection of cells was carried out by the Nanojuice transfection kit (71900-3, Merck, Kenilworth, NJ, USA) following the manufacturer’s instructions.

### 2.4. Motility Assays

Wound healing assay was carried out as described previously [37] with cells 24 h post-transfection in 12-well plates for a duration of 12 h. The represented results are the average of three repetitions, and statistical significance was determined using the Student’s *t*-test. Transwell assay was carried out as described previously [40] with the following modifications: cells 24 h post-transfection were plated in plate filters with pores of 5 μm (3421, Corning, Corning, NY, USA). Four hours after plating the membranes were fixed for 10 min in 2% paraformaldehyde-PBS. Cells were permeabilized by 0.1% Triton X-100 in PBS and nuclei stained with Hoechst. After releasing the filters from the inserts, only transfected cells were counted. The represented results are the average of three repetitions, and statistical significance was determined using the Student’s *t*-test.

### 2.5. Immunostaining

Cells plated on fibronectin-coated coverslips (03-090-1-05, Biological Industries, Beit-Haemek, Israel) and grown to confluence were scratched for the wound healing assay. After the scratch, the cells were further incubated at growth conditions for 3 h. The cells were fixed in 2% paraformaldehyde for 10 min at room temperature. Antibodies used were polyclonal goat anti-lamin B (6216, Santa Cruz Biotechnology, Dallas, TX, USA). Actin filaments were labeled by DyLight™ 554 Phalloidin (13054, Cell Signaling Technologies, Danvers, MA, USA). DNA was stained with Hoechst 33,342 (B2261, Sigma-Aldrich, Rehovot, Israel). Images were collected using an Olympus IX81 fluorescence microscope with a coolSNAP HQ2 CCD camera (Photometrics, Tuscon AZ, USA) and a Zeiss LSM 700 laser (Carl Zeiss Meditec Group, Jena, Germany) scanning confocal microscope. The ImageJ program version 1.52 (NIH) was used to measure Phalloidin mean intensities at the nuclear envelopes, as marked by lamin B immunostaining or GFP signal in cells transfected with GFP-Lamin A or GFP-Lamin B1.

For 3D culture, cells were embedded in 1.7 mg/mL Collagen type I from rat tail (ALX-522-435-0100, Enzo): 80 μL of a solution containing 3 × 10^4^ cells, 1x MEM, 3 mg/mL sodium bicarbonate, 2 mM Glutamine, and 1.7 mg/mL Collagen type I were spread over a cover glass in a 6-well plate and incubated at 37 °C, 7% CO_2_ for 80 min to let the collagen to set. After adding 2 mL of growth medium, the cells were incubated overnight before fixation in 2% paraformaldehyde for 10 min at room temperature. Staining was done as described above.

### 2.6. Western Blot Analysis

Transfected B16–F10 cells were collected by scraping. Cell lysates were prepared by sonication in 2 X SDS sample buffer (100 mM Tris 6.8, 2% SDS, 10% glycerol, 100 mM DTT) supplemented with protease inhibitor cocktail (539134, Merck, Kenilworth, NJ, USA) followed by heating to 95 °C for 10 min. To compare control to migrating cells, cells plated on fibronectin-coated plates were grown to confluence. Migration was induced by multiple scratches at a single plate as described before [41]. After one washing with DMEM, the cells were incubated in a growth medium at 37 °C and 7% CO_2_ for 3 h. Control cells were kept in similar conditions without being scratched. The proteins were separated by SDS–PAGE and transferred to nitrocellulose membranes. The following antibodies were used for Western blotting: goat anti GFP (ab5450, Abcam, Cambridge, MA, USA), rabbit anti lamin B (ab16048, Abcam, Cambridge, MA, USA), mouse anti lamin A/C (4777, Cell Signaling Technology, Danvers, MA, USA), and rabbit anti CTCF (3418, Cell Signaling Technology, Danvers, MA, USA).

## 3. Results

### 3.1. Lamin B1 Over-Expression Elevates the Migration Rate of Melanoma Cells

**Figure 1:**
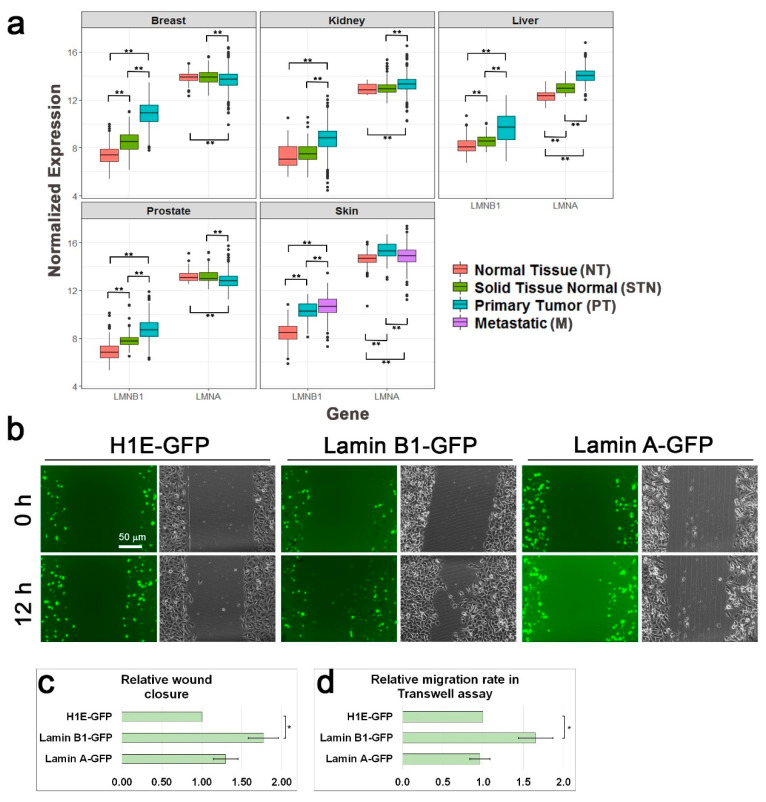
Increased lamin B1 levels in cancer accelerate cell migration. (**a**) Cancer is associated with higher expression levels of lamin B1. Relative RNA levels of lamin A (*LMNA* gene) and lamin B1 (*LMNB1* gene) in the indicated human organs in normal tissues (NT), normal tissues around a tumor (STN), primary tumors (PT), and metastases (M) according to the available data in GTEx and TCGA databases. Patients no.: breast, NT-179, STN-113, PT-1092; kidney, NT-27, STN-140, PT-884; liver, NT-110, STN-50, PT-369; prostate, NT-100, STN-52, PT-495; skin, NT-556, PT-102, M-366; Statistical significance was evaluated by the Student’s *t*-test, ** *p* < 0.01 (**b**) Relative migration rate of B16–F10 cells over-expressing GFP-fused histone H1E, lamin A and lamin B1 in the wound healing assay. Representative GFP and phase-contrast micrographs of the same fields at time 0 (immediately after the scratch) and 12 h later. Scale bar: 50 μm. (**c**) The area covered by the cells following 12 h of incubation was measured and set as 1 for the over-expressing H1E-GFP cells. The graphs show the mean area covered in five independent experiments ± s.e. Statistical significance was evaluated by the Student’s *t*-test, * *p* < 0.05. (**d**) Over-expression of lamin B1 accelerates the migration rate of B16–F10 in the Transwell assay. The migration rate of B16–F10 cells over-expressing H1E-GFP, lamin B1-GFP, and lamin A-GFP was measured by the Transwell assay. Four hours after plating the cells on top of the filters the cells were fixed, permeabilized, and stained with Hoechst reagent. In each experiment the fraction of the transfected cells migrated to the lower side of the filter was calculated and normalized to the migration rate of H1E-GFP over-expressing cells. The graphs show the mean relative migration rate in three independent experiments ± s.e. Statistical significance was evaluated by the Student’s *t*-test, * *p* < 0.05.

Lamin B1 has been found to be important for cell migration of several cell types [12,13,15,17]. Enhanced migration rate is one of the cancer hallmarks [42], therefore, we compared lamin B1 and lamin A expression levels between normal cells and tumor cells as well as between primary tumors and their metastases using GTEx (for normal tissues) and TCGA datasets. As shown in Figure 1a, lamin B1 RNA levels are significantly higher in various types of cancer including breast, kidney, liver, prostate, and skin. In addition, in skin cancer samples, lamin B1 RNA levels are higher in metastatic tumor samples than in primary tumor samples. On the contrary, lamin A RNA levels are upregulated in some types of cancer while down-regulated in others. To evaluate the effects of lamin B1 and lamin A on the migration rate of melanoma cells, we over-expressed these two genes in B16–F10 cells and measured the cellular migration rate. As a control, we used B16–F10 cells over-expressing histone H1E, which we previously found to not affect cell migration rate [40]. As expected, migration rate of lamin B1 over-expressing cells was increased by more than 50% in comparison to control cells over-expressing histone H1E in both the wound healing assay and the Transwell assay. On the other hand, lamin A over-expression did not change the cellular migration rate in a significant manner (Figure 1b–d), although the over-expression of lamin A was to a higher extent than the over-expression of lamin B1 (Figure S1).

### 3.2. Migration Signals Induce the Formation of a Perinuclear Actin Rim

**Figure 2:**
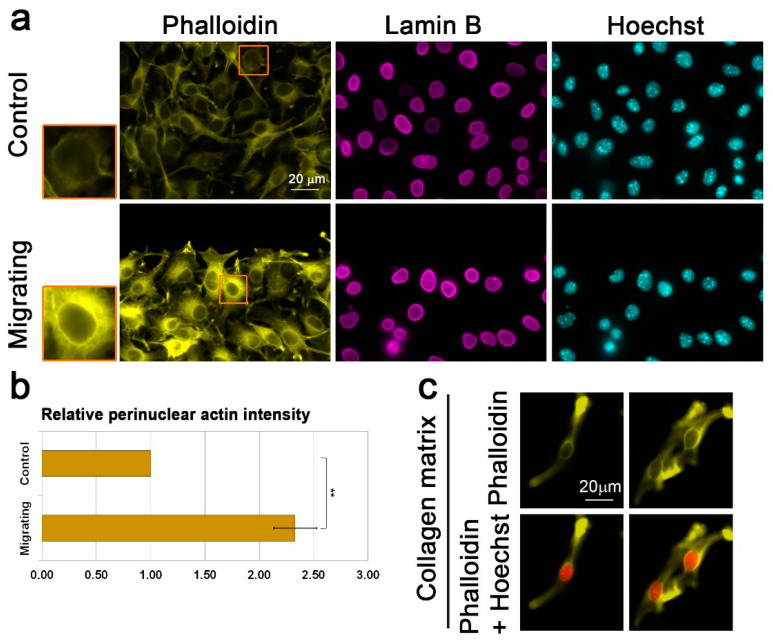
Migration signals induce the formation of a perinuclear actin rim. (**a**) Perinuclear actin rim in B16–F10 migrating in the wound healing assay. Fluorescence microscope micrographs of confluent B16–F10 cells induced to migrate in the wound healing assay for 3 h stained for filamentous actin (Phalloidin) and DNA (Hoechst). Lamin B1 was immunostained. The edge of the scratch is in the top region of each micrograph. Scale bar: 20 μm. The nuclei in the orange rectangles are magnified on the left side. (**b**) Quantification of the actin perinuclear rim in B16–F10 migrating in the wound healing assay. The bar graph represents the change in intensity of perinuclear Phalloidin staining in migrating cells in comparison to control non-migrating cells. For quantification, in each experiment, 30–60 cells of each condition were measured, and the average mean intensity was calculated and normalized to control cells. The average mean intensity in three independent experiments ± s.e. is presented. Statistical significance was evaluated by the Student’s *t*-test, ** *p* < 0.01. (**c**) Collagen matrix induces accumulation of perinuclear actin filaments in B16–F10 cells. Fluorescence microscope micrographs of B16–F10 cells embedded inside collagen matrix for O.N. stained for filamentous actin (Phalloidin) and DNA (Hoechst). Two representative images are shown.

The mechanism by which lamin B1 enhances the cellular migration rate is thought to be due to different biomechanical properties of the B-type lamin network than an A-type lamin network [19,25,26,27,28,29]. We hypothesize that in addition to that, the two different types of lamins may affect differently the cytoskeleton organization around the nucleus, leading to different effects on the cellular migration capabilities. To test this hypothesis, we looked for the organization of actin filaments around the nucleus of migrating cells. Significantly, B16–F10 cells induced to migrate in the wound healing assay for 3 h acquired a perinuclear actin rim that was hardly detected in non-migrating cells (Figure 2a,b). The rim appeared already 30 min after induction of migration (Figure S2). This actin rim seemed to form at the outer side of the nucleus according to a localization profile analysis of images taken by a laser scanning confocal microscope (Figure S3). Such a perinuclear actin rim has not been reported before in migrating cells, but a transient perinuclear actin rim that lasted for only a few minutes (up to 5 min) was detected in cells that were stimulated by mechanical force or ATP [43,44,45]. To test if a perinuclear actin rim is found in cells in a 3D environment, we embedded the B16–F10 cells in a collagen gel. As seen in Figure 2c, this rim was seen also in elongated cells embedded in collagen gel, suggesting that the perinuclear actin rim is a feature of migrating cells in both 2D and 3D environments.

### 3.3. Lamin B1 Over-Expression Interferes with Perinuclear Actin Rim Formation

**Figure 3:**
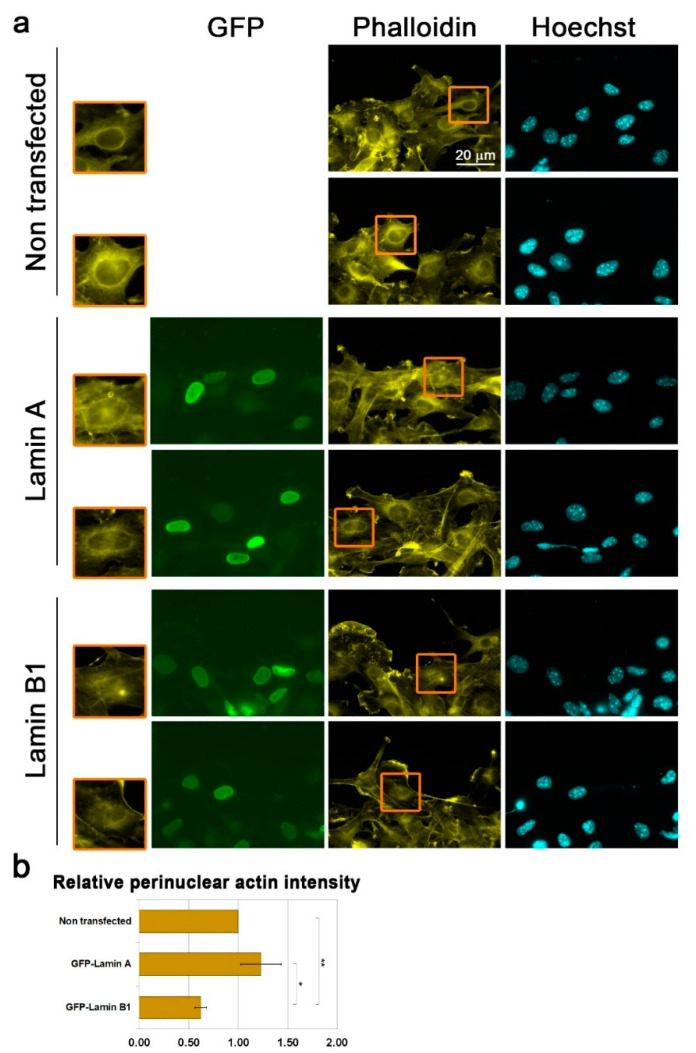
Increased lamin B1 levels interfere with the perinuclear actin rim levels. (**a**) Fluorescence microscope micrographs of non-transfected and over-expressing GFP-fused lamin A or GFP-fused lamin B1 confluent B16–F10 cells induced to migrate in the wound healing assay for 3 h stained for filamentous actin (Phalloidin) and DNA (Hoechst). The edge of the scratch is in the top region of each micrograph. The nuclei in the orange rectangles are magnified on the left side. Scale bar: 20 μm. (**b**) Quantification of the effect of lamins over-expression on perinuclear actin filaments. In each experiment, 20–40 cells of each transfection were measured for the Phalloidin signal at the nuclear periphery. The average mean intensity was calculated and normalized to non-transfected cells. The average mean intensity in three independent experiments ± s.e. is presented. Statistical significance was evaluated by the Student’s *t*-test, * *p* < 0.05, ** *p* < 0.01.

Next, we examined if over-expression of lamin B1 and lamin A affects the migration-associated perinuclear actin rim. Significantly, whereas the perinuclear actin rim was present in lamin A over-expressing cells, it was reduced by almost 50% in lamin B1 over-expressing cells (Figure 3). Nuclei of cells with very high over-expression levels of lamin B1 were distorted. Although these cells did not have a perinuclear actin rim, we did not add them to our analysis. In agreement with the negative effect of lamin B1 on perinuclear actin rim formation, the levels of lamin B1 were not increased in B16–F10 upon induction of migration (Figure S4). In summary, lamin B1 enriched lamina can interfere with cytoplasmic actin filaments adjacent to the nuclear envelope. 

### 3.4. Interference with Perinuclear Actin Filaments Increases the Cellular Migration Rate

**Figure 4:**
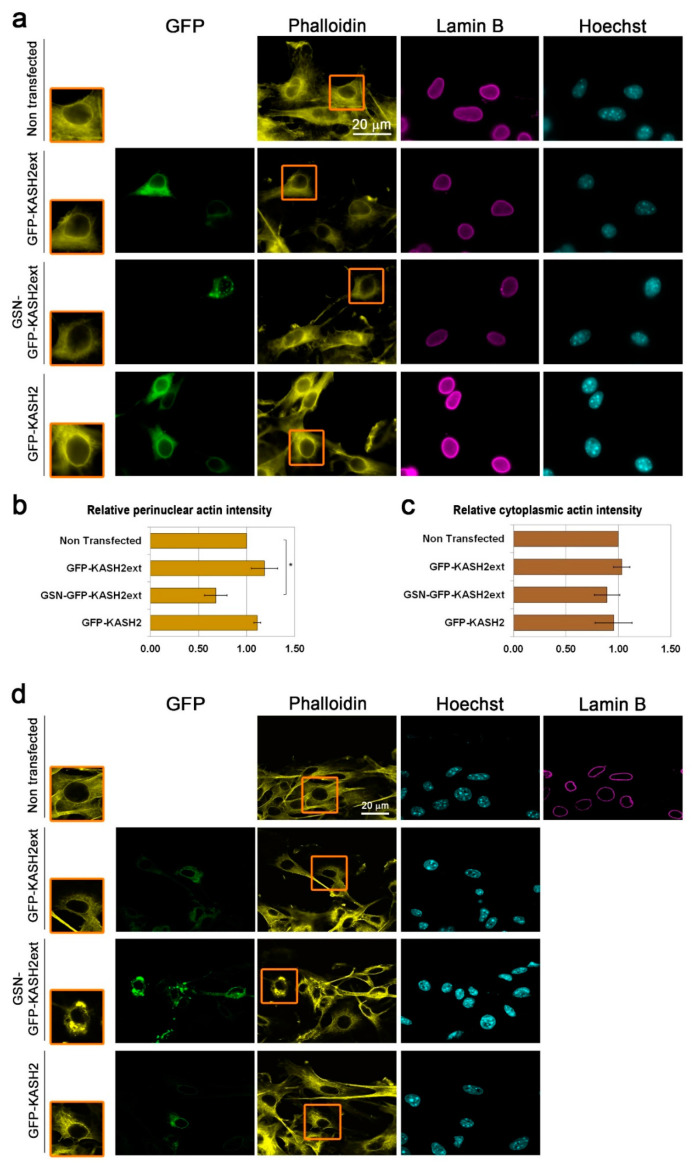
Interference with the perinuclear actin rim by perinuclear-localized Gelsolin (GSN) (**a**) Fluorescence microscope micrographs of confluent B16–F10 cells either non-transfected or expressing GFP-KASH2ext, GSN-GFP-KASH2ext, or GFP-KASH2 induced to migrate in the wound healing assay for 3 h stained for filamentous actin (Phalloidin) and DNA (Hoechst). Lamin B1 was immunostained. The edge of the scratch is in the top region of each micrograph. The nuclei in the orange rectangles are magnified on the left side. Scale bar: 20 μm. (**b**) Quantification of the effect of GFP-KASH2ext, GSN-GFP-KASH2ext, and GFP-KASH2 on perinuclear actin filaments. In each experiment, 20–30 cells of each transfection were measured for the Phalloidin signal at the nuclear periphery. The average mean intensity was calculated and normalized to non-transfected cells. The average mean intensity in three independent experiments ± s.e. is presented. Statistical significance was evaluated by the Student’s *t*-test, * *p* < 0.05. (**c**) Quantification of the effect of GFP-KASH2ext, GSN-GFP-KASH2ext, and GFP-KASH2 on the total amount of actin filaments. In each experiment, 20–30 cells of each transfection were measured for the mean Phalloidin signal in the whole cell. The average mean intensity was calculated and normalized to non- transfected cells. The average mean intensity in three independent experiments ± s.e. is presented. Differences between samples were not significant statistically by the Student’s *t*-test (**d**) Laser scanning microscope micrographs of non-transfected and transfected B16–F10 cells as in a.

Perinuclear actin structures such as the LINC complex-dependent actin cap and TAN lines were shown to affect the cellular migration capabilities [31,33,46,47], therefore, we wanted to evaluate the effect of the perinuclear actin rim on the cellular migration rate. To do so, depolymerization of it would be required, however, disruption of the perinuclear actin filaments with a general actin depolymerization drug such as cytochalasin D will lead to depolymerization of most other actin filaments in the cell that are crucial for proper cellular migration. Therefore, we developed a specific chimeric protein that disrupts actin filaments, which are in close proximity to the nucleus. For that purpose, we used the GFP fused KASH2ext peptide, which is a modified fragment from Nesprin 2. GFP-KASH2ext was shown to localize to the outer nuclear membrane without affecting the LINC complex [39]. We fused it to a constitutive form of the actin severing protein GSN, which lacks the auto-inhibitory regulatory domains. As seen in Figure 4, expression of GFP-KASH2ext did not interfere with the appearance of the perinuclear actin rim in migrating cells as analyzed by both fluorescent microscope and laser scanning microscope. On the other hand, expression of GSN-GFP-KASH2ext did interfere with perinuclear actin filaments: it reduced their levels and led to the disappearance of their organization in a defined rim-like structure without affecting the levels of lamin B1 while many other cytoplasmic actin filaments were still present in these cells (Figure 4 and Figure S5). Interestingly, over-expression of GFP-KASH2, which is an inhibitor of the endogenous LINC complex [39], did not interfere with the formation of the perinuclear actin rim in migrating cells (Figure 4a,b, Figure S5), suggesting that the LINC complex is not required for the perinuclear actin rim formation in migrating cells.

**Figure 5:**
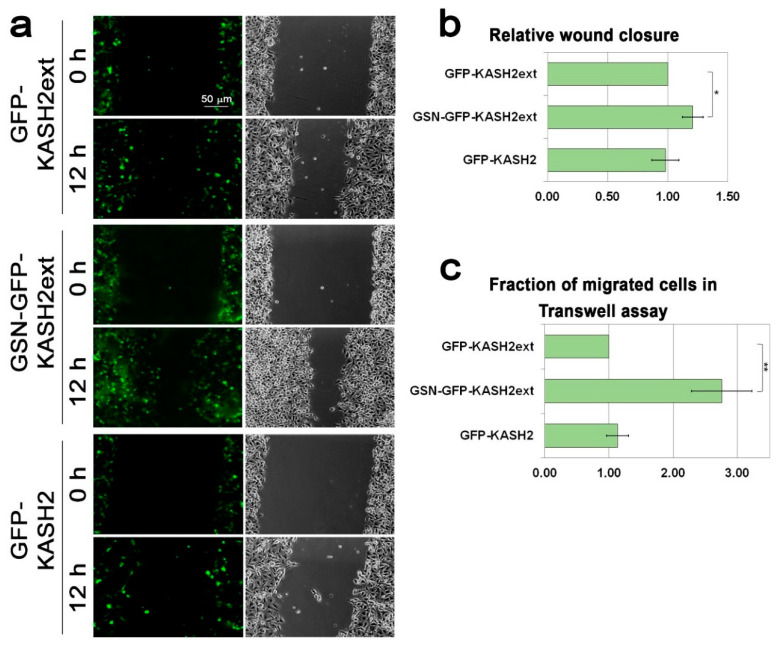
Interference with the perinuclear actin rim accelerates cell migration rate. (**a**) Interference with perinuclear actin filaments accelerates the migration rate of B16–F10 in the wound healing assay. The migration rate of B16–F10 cells expressing GFP-KASH2ext, GSN-GFP-KAS2ext, or GFP-KASH2 was measured by the wound healing assay. Representative GFP and phase-contrast micrographs of the same fields at time 0 (immediately after the scratch) and 12 h later. Scale bar: 50 μm. (**b**) The area covered by the cells following 12 h of incubation was measured and set as 1 for the expressing GFP-KASH2ext cells. The graphs show the mean area covered in three independent experiments ± s.e. Statistical significance was evaluated by the Student’s *t*-test, * *p* < 0.05. (**c**) Interference with perinuclear actin filaments accelerates the migration rate of B16–F10 in the Transwell assay. The migration rate of B16–F10 cells expressing GFP-KASH2ext, GSN-GFP-KAS2ext, or GFP-KASH2 was measured by the Transwell assay. Four hours after plating the cells on top of the filters the cells were fixed, permeabilized, and stained with Hoechst reagent. In each experiment, the fraction of the transfected cells migrated to the lower side of the filter was calculated and normalized to the migration rate of GFP-KASH2ext-expressing cells. The graphs show the mean fraction of migrated cells in three independent experiments ± s.e. Statistical significance was evaluated by the Student’s *t*-test, ** *p* < 0.01.

Next, we evaluated the effect of GSN-GFP-KASH2 on cell migration rate. Significantly, in the wound healing assay, GSN-GFP-KASH2ext expressing cells migrated 1.2-fold faster than GFP-KASH2ext expressing cells, while interference with the LINC complex by expression of GFP-KASH2 did not affect the migration rate of the cells (Figure 5a,b). The positive effect of GSN-GFP-KASH2ext expression on cell migration rate was increased to 2.7-fold once the migration rate was evaluated by the Transwell assay that requires bigger changes in nuclear morphology than the wound healing assay (Figure 5c). Interestingly, even in this migration assay, expression of GFP-KASH2 did not affect the migration rate of the cells, suggesting that migration of mouse melanoma cells is LINC complex-independent. On the contrary, migration of these cells was accelerated by interfering directly with the perinuclear actin rim (Figure 5) as well as by over-expression of Lamin B1 (Figure 1) that interfered with the perinuclear actin rim (Figure 3). Overall, our results support a model in which increased lamin B1 levels can accelerate the cellular migration rate by interfering with perinuclear actin rim formation.

## 4. Discussion

Our results suggest that increased expression of lamin B1 can promote cell migration at least partially by inhibiting the formation of a perinuclear actin rim that normally constrains cell migration rate. A perinuclear actin rim has been found to form for a very short period of time (on a timescale of 1–5 min) in few cell types in response to an external mechanical force or to an increased extracellular ATP concentration [43,44,45]. This polymerization of perinuclear actin filaments was shown to be induced by inverted formin 2 (INF-2) that was activated by increased cytosolic concentration of calcium ions [43,45], but independent of the LINC complex [43]. The perinuclear actin rim we identified in melanoma cells has a much longer lifetime, which is on a timescale of hours (Figure 2 and Figure S2), however, it is also LINC complex-independent (Figure 4). The transient INF-2-dependent perinuclear actin rim was suggested to control transcription by inducing translocation of the transcription coactivator Myocardin related transcription factor-A (MRTF-A) from the cytoplasm to the nucleus and to enhance migration rate of cells [45]. However, manipulations of the perinuclear actin rim formation in the previous study were done by either knocking down INF-2, induction of calcium ions influx, or addition of ATP to the cell medium [45], methods that in addition to affecting perinuclear actin filaments may affect additional aspects and factors in the cell. Here, we generated a new tool to disrupt the defined organization of perinuclear actin filaments in a rim-like structure by fusing the GSN catalytic domain to KASH2ext. This tool enabled us to show that perinuclear actin rim constrains cell migration rate (Figure 5). 

Notably, perinuclear actin filaments are formed in response to migration signals, although they inhibit the cellular migration rate. Thus, these filaments should have some roles in migrating cells. We hypothesize that these perinuclear actin filaments protect the nucleus from possible mechanical insults during migration processes. This hypothesis is supported by previous studies showing that increased levels of B-type lamins reduced nuclear rigidity while increased nuclear fragility. The proposed mechanism for that was based on different mechanical properties of the different networks formed by B-type and A-type lamins [19,24,25,26,27,28,29]. In addition, lamin A was suggested to support actin cap formation in mouse embryonic fibroblasts and C2C12 cells [47,48]. Our data add to this explanation the ability of B-type lamins rather than A-type lamins to interfere with the organization of perinuclear actin filaments. 

The ability of lamin B1 to affect actin dynamics outside of the nucleus suggests an inside-out mechanism between the inner side and the outer side of the nuclear envelope. One possible component to support such communication is the LINC complex [49]. However, we found that the LINC complex is not required for the perinuclear actin formation in B16–F10 cells (Figure 4) and it does not support migration of these cells (Figure 5). Our results are in agreement with previous findings showing that the LINC complex is dispensable for NIH3T3 perinuclear actin rim formation [43] and that components of the LINC complex are down-regulated in cancer [50,51,52,53,54,55]. Thus, we estimate that an additional mechanism to the LINC complex links the nuclear envelope to the cytoplasmic actin cytoskeleton, a mechanism that is sensitive to high levels of lamin B1. 

## Supplementary Materials

The following are available online at https://www.mdpi.com/2073-4409/9/10/2161/s1, Figure S1: Levels of over-expressed lamin A and lamin B1. Figure S2: Migration signals induce the formation of a perinuclear actin rim. Figure S3: Migration induced perinuclear actin rim is peripheral to the nuclear lamina. Figure S4: Induction of migration does not alter the protein levels of lamin B1 and lamin A. Figure S5: Levels of expressed GSN-GFP-KASH2ext, GFP-KASH2ext and GFP-KASH2.
