# Supplementary material for: Increased Lamin B1 Levels Promote Cell Migration by Altering Perinuclear Actin Organization
Source: Cells. 2020 Sep 24;9(10):2161. doi: 10.3390/cells9102161 (PMC7598699; doi:10.3390/cells9102161)
Supplement: Supplementary file 1 [file cells-09-02161-s001.pdf]

Increased lamin B1 levels promote cell migration by altering perinuclear actin organization

Andrea Fracchia, Tal Asraf, Mali Salmon-Divon and Gabi Gerlitz*

Department of Molecular Biology, Faculty of Life Sciences and Ariel Center for Applied Cancer Research, Ariel University, Ariel 40700, Israel.

*Correspondence to: Department of Molecular Biology, Faculty of Natural Sciences, Ariel University, Kiryat Hamada, Ariel 40700, Israel. E-mail addresses: gabige@ariel.ac.il

Supplementary figures

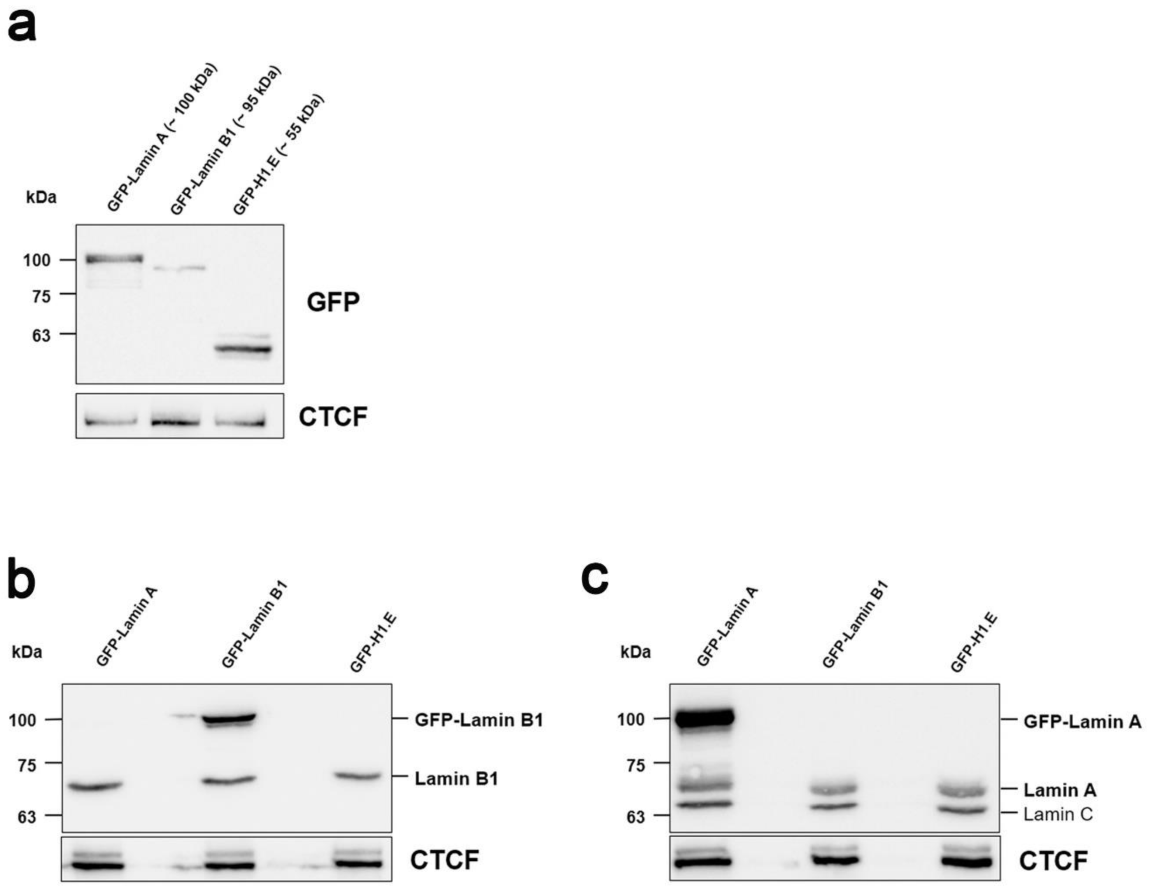

Figure S1 Levels of over-expressed lamin A and lamin B1. (**a**) Western blot analysis of the relative levels of over-expressed lamin A, lamin B1 and histone H1E in B16-F10 cells using antibodies against GFP. CTCF was used as a loading control. (**b**) Western blot analysis of the relative lamin B1 levels in B16-F10 cells over-expressing GFP-fused lamin A, lamin B1 and histone H1E using antibodies against lamin B. CTCF was used as a loading control. (**c**) Western blot analysis of the relative lamin A levels in B16-F10 cells over-expressing GFP-fused lamin A, lamin B1 and histone H1E using antibodies against lamin A/C. CTCF was used as a loading control.

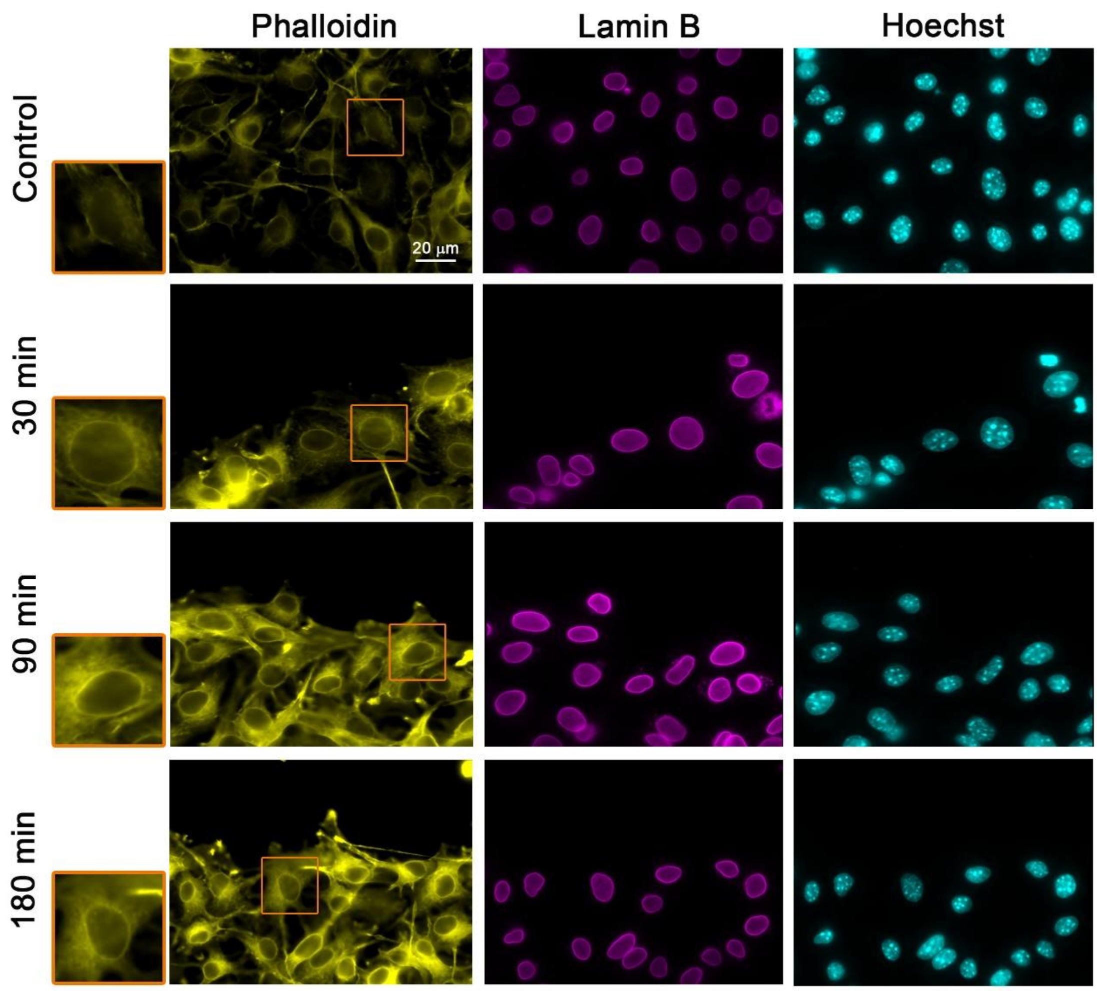

Figure S2 Migration signals induce the formation of a perinuclear actin rim. Fluorescence microscope micrographs of confluent B16-F10 cells induced to migrate for various time points in the wound healing assay stained for filamentous actin (Phalloidin) and DNA (Hoechst). Lamin B1 was immunostained. The time points are listed to the left of the micrographs. The edge of the scratch is in the top region of each micrograph. Scale bar: 20 µm. The nuclei in the orange rectangles are magnified at the left side.

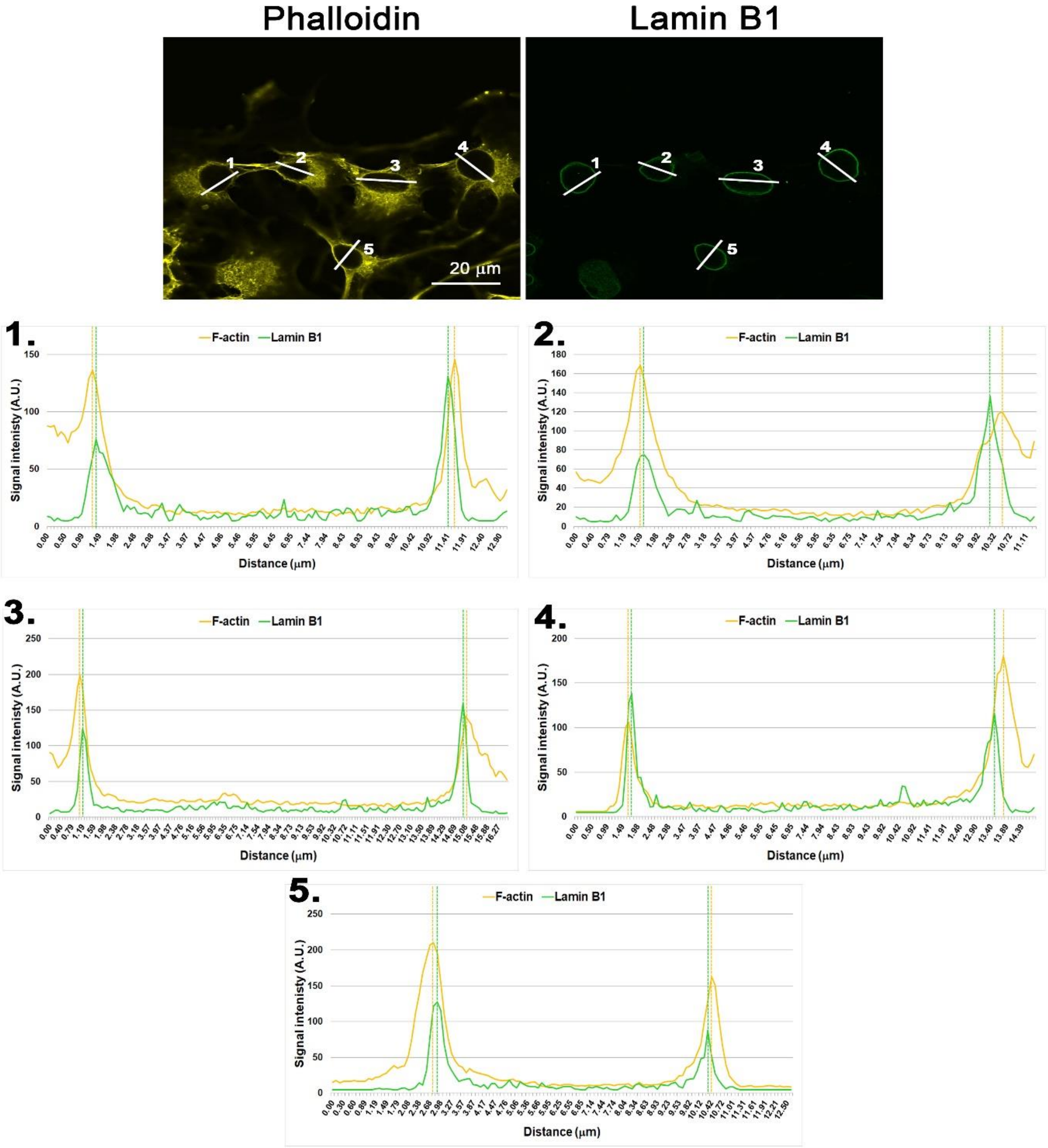

Figure S3 Migration induced perinuclear actin rim is peripheral to the nuclear lamina. Localization profile of actin filaments (Phalloidin staining) and nuclear lamina (immunostaining of lamin B) in migrating B16-F10 cells in the wound healing assay in a micrograph taken by laser scanning microscope. Scale bar: 20 µm.

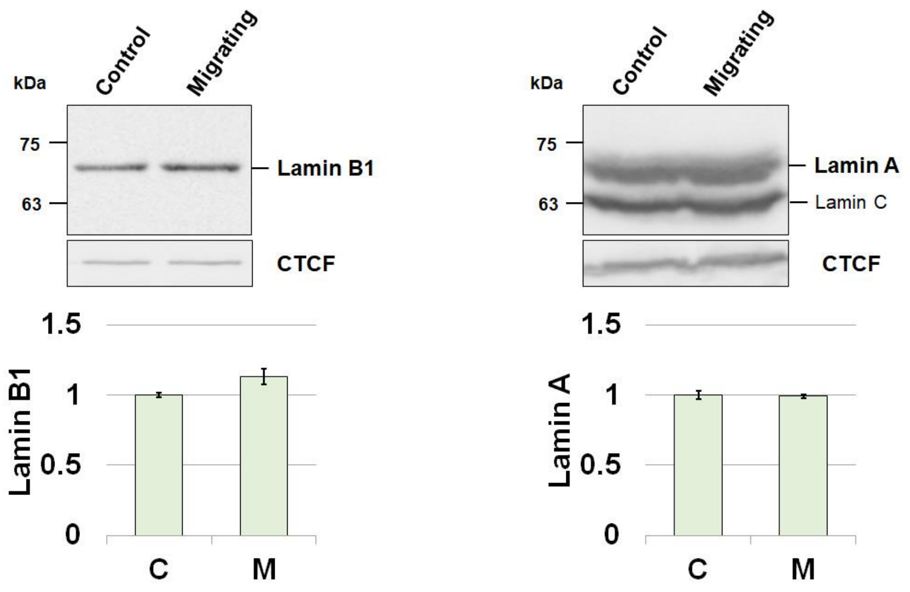

Figure S4 Induction of migration does not alter the protein levels of lamin B1 and lamin A. Western blot analysis of lamin B1 and lamin A protein levels in control and migrating B16-F10 cells. CTCF was used as a loading control. The bar graphs represent the means of lamin B1 and lamin A levels that were normalized to CTCF levels ± s.e. of three independent experiments.

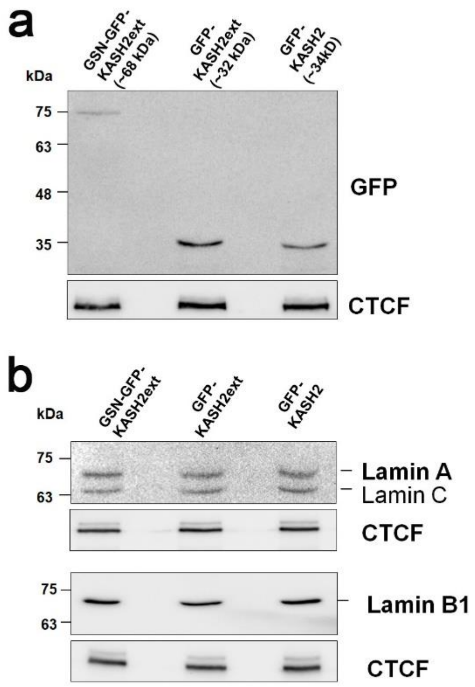

Figure S5 Levels of expressed GSN-GFP-KASH2ext, GFP-KASH2ext and GFP-KASH2. (a) Western blot analysis of the relative levels of GSN-GFP-KASH2ext, GFP-KASH2ext and GFP-KASH2 in B16-F10 cells using antibodies against GFP. CTCF was used as a loading control. (b) Western blot analysis of the relative levels of lamin A and lamin B1 in B16-F10 cells transfected with GSN-GFP-KASH2ext, GFP-KASH2ext and GFP-KASH2. CTCF was used as a loading control.
